# Combination treatment of pembrolizumab with DC-CIK cell therapy for advanced hepatocellular carcinoma: A case report

**DOI:** 10.37796/2211-8039.1414

**Published:** 2023-09-01

**Authors:** Shao M. Huang, Long-Bin Jeng, Woei-Cherng Shyu, Hung-Yao Chen

**Affiliations:** a China Medical University Hospital, Taichung, Taiwan; b Division of Hepatogastroenterology, Department of Internal Medicine, China Medical University Hospital, Taichung, Taiwan; c Translational Medicine Research Center, Drug Development Center and Department of Neurology, China Medical University Hospital, Taichung, Taiwan; d Graduate Institute of Biomedical Science and Drug Development Center, China Medical University, Taichung, Taiwan; e Department of Occupational Therapy, Asia University, Taichung, Taiwan; f Organ Transplantation Center, China Medical University Hospital, Taichung, Taiwan

**Keywords:** Hepatocellular carcinoma, DC-CIK, Immune checkpoint inhibitor, Pembrolizumab, Combination therapy

## Abstract

**Background:**

Recently, immunotherapy has emerged as a promising method for advanced HCC treatment. There are several clinical trials and meta-analyses of immune checkpoint inhibitors and immune cell therapy, but clinical evidence on the combination of these two therapies is lacking.

**Case description:**

A 66-year-old man with chronic hepatitis B-related cirrhosis complained of acute abdominal pain in an emergency department of a hospital. On exams, there was a palpable mass in the right upper quadrant of his abdomen. Contrast-enhanced abdominal computed tomography showed a large tumor in the right lobe, 13 cm × 17 cm in size, and right portal vein thrombosis. The alpha-fetoprotein (AFP) level was 30,905 mg/dL. Therefore this patient was diagnosed with BCLC stage C hepatocellular carcinoma (HCC). He underwent trans-arterial chemo-embolization (TACE), abdominal radiotherapy, nivolumab, and lenvatinib. His disease had been under control until two years later, the disease progressed with multiple lung metastases, and his AFP level rose from around 1000 to 17,000 ng/ml. At this stage, he underwent new combination immunotherapy in January 2022. He used pembrolizumab (100 mg) first, and the AFP level decreased by 600 ng/ml daily. Then he received DC-CIK cell therapy two weeks after using pembrolizumab, and the AFP level declined to 900 ng/ml a day. Unfortunately, severe pneumonitis and tension pneumothorax developed after therapy. The patient denied undergoing further treatment and expired peacefully.

**Conclusion:**

The previous in-vivo study found that combination immunotherapy can improve tumor control in the mice model. Besides, in previous clinical studies, the level of AFP may be a surrogate marker of tumor response. Therefore we thought the more rapidly declined level of AFP was the clinical evidence of the synergistic effect of checkpoint inhibitors combined with cell therapy in HCC treatment.

## 1. Introduction

According to WHO 2020 statistics, primary liver cancer is the sixth most commonly diagnosed cancer and the third leading cause of cancer death worldwide [1]. Approximately 75 percent of primary liver tumors are Hepatocellular Carcinoma (HCC), which usually develops from chronic liver diseases, such as chronic hepatitis B or C, alcoholic or nonalcohol-associated steatohepatitis (NASH) related cirrhosis 1. For HCC treatment, surgery and locoregional therapies are considered for early-stage HCC, while multi-kinase inhibitors and immune checkpoint inhibitors are preferred for advanced HCC. In recent years, immunotherapy, including checkpoint inhibitors, immune cell therapy, and cancer vaccine, has emerged as a promising therapy for advanced HCC. National Comprehensive Cancer Network (NCCN) recommends using Atezolizumab (PD-L1 inhibitor) and bevacizumab (VEGF inhibitor) as the first-line therapy to treat advanced HCC. This regimen significantly improved 12-month overall survival (OS) and median progression-free survival (PFS) compared to sorafenib in the IMbrave150 study [2]. As to immune cell therapy, peripheral blood mononuclear cells (PBMC) that express the CD3 and CD56 molecules simultaneously have been proven to have the highest killing potential against tumor cells in vitro since 1991 [19]. In the past ten years, CIK cell-based immunotherapy has demonstrated considerable cytotoxicity against various malignancies [20]. Many metaanalyses show immune cell therapy significantly improves overall survival and significantly reduces the recurrence rate in HCC patients [3]. Checkpoint inhibitors and immune cell therapy have already been evaluated individually for their clinical efficacy on HCC. However, only one case report [4] discussed the effect of immune checkpoint inhibitors in combination with immune cell therapy to treat HCC.

This report describes a case report of advanced HCC treated with an immune checkpoint inhibitor followed by a DC-CIK vaccine.

We present the following case following the CARE reporting checklist.

## 2. Case report

A 66-year-old man with a history of chronic hepatitis B presented to the emergency department with acute right-upper-quadrant (RUQ)abdominal pain for hours. On examinations, his abdomen was soft but tender to deep palpation at RUQ with a palpable mass. His abdominal Computed Tomography (CT) showed a substantial hypervascular tumor in the right lobe (13 × 17 cm in size) and thrombosis in the right portal vein. The level of Alpha-fetoprotein (AFP) at that time was as high as 30,905 ng/ml. He was diagnosed with HCC, BCLC, stage C.

**Fig. 1:**
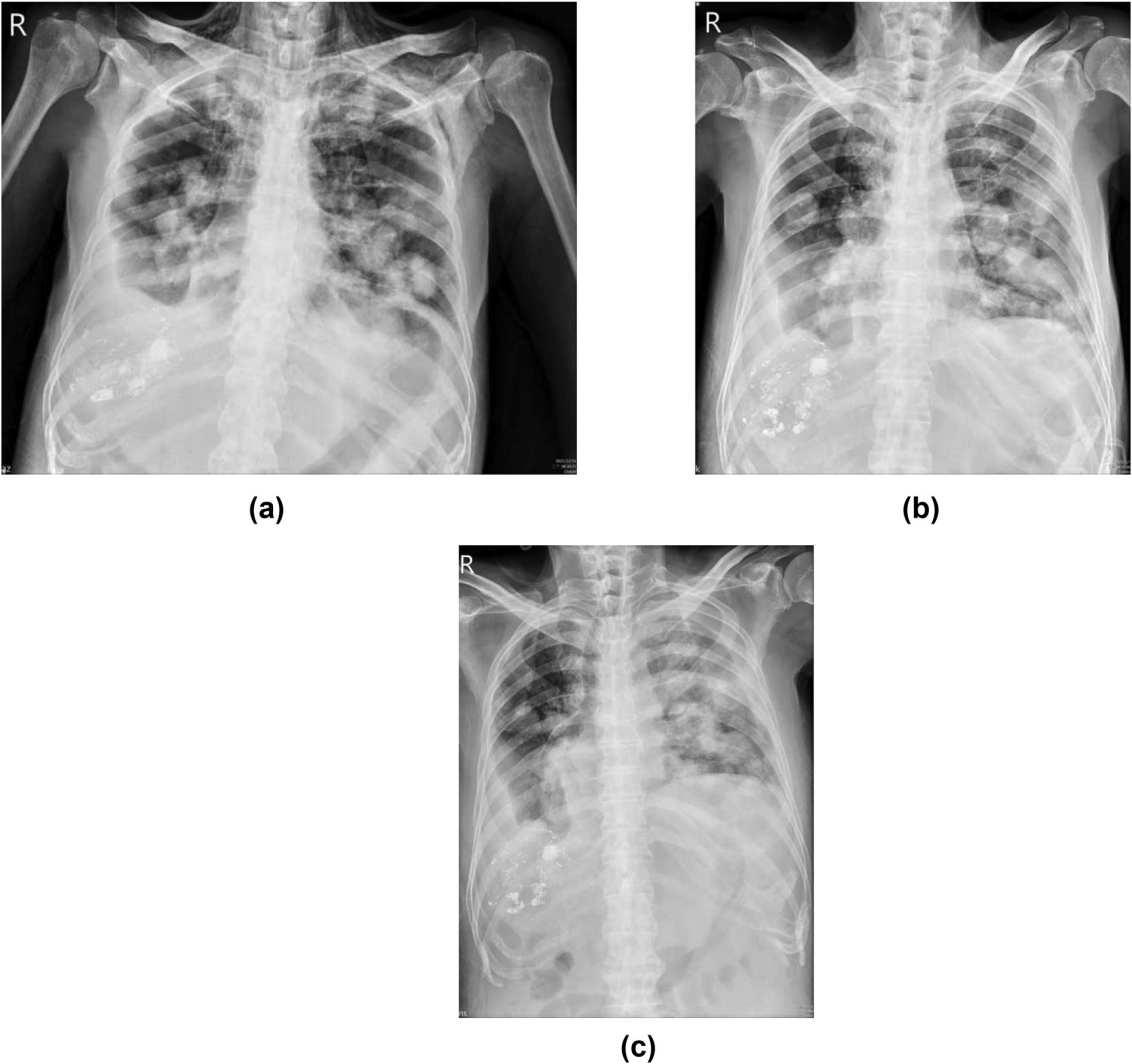
a. The anteroposterior (AP) view of the patient’s chest X-ray film on 31 December 2021. Before using pembrolizumab, multiple metastatic tumors were in both lungs, and the border of each tumor was sharp and distinct. Bilateral pleural effusions were noted. Besides, the patient also had subcutaneous emphysema in the lower neck and upper chest wall, a complication of radiotherapy to his mediastinal lymphadenopathy in Dec 2021. b. AP view of the patient’s chest X-ray film on 7 January 2022. The border of metastatic tumors became blurred with increasing infiltration. This phenomenon might be due to the clearance of HCC by T cells and inducing inflammatory reactions. c. AP view of the patient’s chest X-ray film on 14 January 2022. The infiltration area, especially in the left lung, became more prominent than that one week ago. There was no apparent ground glass pattern in the right upper lobe. This phenomenon might be because there were no evident metastatic nodules in this area. Ten days later, left tension pneumothorax developed. This event might result from the necrosis of metastatic tumors in the left lung.

Since then, he has received three courses of transarterial chemoembolization (TACE), 25 times abdominal radiotherapy, and four times intravenous nivolumab 100 mg. After then, he took lenvatinib 10 mg/day. The AFP level was maintained between 1000 and 2000 ng/ml for four months until he discontinued lenvatinib. The AFP level rose quickly to 17,828 ng/ml after lenvatinib discontinuation, and the contrast-enhanced chest CT showed multiple round nodules in his lungs. Due to disease progression, we discussed DC-CIK cell therapy with the patient, and he agreed to try it. Therefore he underwent wedge resection of the left upper and lower lobes for cell therapy preparation using video-assisted thoracic surgery (VATS). Progressive shortness of breath developed four weeks after the left lung wedge resection. The chest CT showed that enlarged mediastinal lymph nodes led to his mild bronchial stenosis. So we first resumed lenvatinib 10 mg/day, followed by radiotherapy to control his lymphadenopathy. Ten days after radiotherapy, we added intravenous pembrolizumab 100 mg, and the AFP level was 16,576 ng/mL. Thirteen days later, we infused 10^9^ DC-CIK cells into the patient. The AFP level was down to 10,570 ng/mL on the same day. One day after DC-CIK cell infusion, shortness of breath developed, and his chest radiograph (CXR) showed increased infiltration in the left lung [Fig. 1ac]. Immune-related adverse events (IRAE) should be considered, and intravenous methylprednisolone 20 mg/day was given two days after the first administration of DC-CIK cells. Before using the steroid, the AFP level decreased to 8704 ng/mL. Unfortunately, 11 days after cell therapy, the left tension pneumothorax occurred. The patient refused to undergo further treatment and expired peacefully.

## 3. Discussion

A prior study suggested that at the receptor/ligand level, the PD-1/PD-L1 pathway is crucial to tumor-induced T-cell immune tolerance [5]. PD-1 is presented both on activated T cell [6] and DC cell [7] surfaces, which suppress these cells’ activity, so it is hypothesized that DCCIK cells show a more potent antitumor effect by neutralizing its surface PD-1 molecule [8]. The clinical effect of the combination of PD-1 inhibitor and cell therapy on various cancers was investigated by several studies, such as Nesselhut et al. They found that nivolumab (antiPD1) in combination with the DC vaccine is helpful in pancreatic cancer patients [9]. Also, a completed single-center, open-label, phase I trial examined the combination of pembrolizumab and DC-CIK vaccine in various types of advanced solid tumors [8]. As to HCC, in vivo studies found that DC-CIK pretreated with pembrolizumab had considerably decreased tumor weight and volume in xenograft mice with HCC [10] compared with DCCIK infusion only. Another study found that transplanting DC without PD-1 expression improves tumor control in mice HCC models [11]. An ongoing open-labeled single-arm phase II (NCT04912765) is the only clinical trial on the combination of checkpoint inhibitor plus cell therapy on HCC, registered on clinicaltrial.gov. The trial aims to investigate the effect of the intra-dermal neoantigen DC vaccine combined with intravenous nivolumab in patients with resectable HCC or liver metastases from colorectal cancer. The final result is still pending.

As to HCC treatment response assessment, aside from radiologic assessment using WHO [12] or mRECIST (Modified Response Evaluation Criteria in Solid Tumors) [13] criteria, the study shows AFP level is significantly associated with radiologic response in advanced HCC patients, therefore may serve as a surrogate marker for evaluating HCC treatment response [14].

**Fig. 2:**
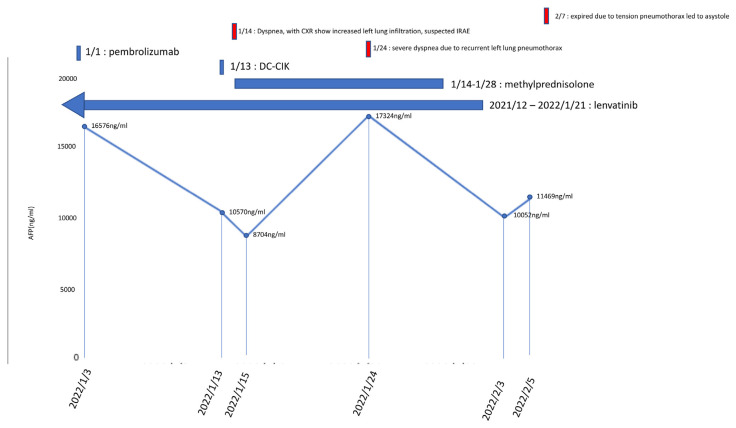
This patient’s alpha-fetoprotein (AFP) levels during the treatment of lenvatinib combined with pembrolizumab and DC-CIK Cells therapy. Intravenous pembrolizumab 100 mg was used on 3 January 2022, and the AFP level was 16,576 ng/mL. Thirteen days later, we added intravenous DC-CIK cell therapy. The AFP level was down to 10,570 ng/mL on the same day. Two days later, IRAE was suspected due to acute shortness of breath and increased infiltration in his chest X-ray film. We started using intravenous methylprednisolone 20 mg/day to terminate this combined immunotherapy on 15 January 2022. Left tension pneumothorax developed on 24 January 2022. days and the patient received percutaneously placed pigtail catheters on the same day and discontinued using the steroid on 28 January 2022. However, his pneumothorax progressed by days under negative pressure drainage. Surgical intervention was indicated, but he refused to undergo any treatment, including air drainage. Therefore we removed his pigtail catheter on 3 February 2022, and he expired peacefully two days later.

**Fig. 3:**
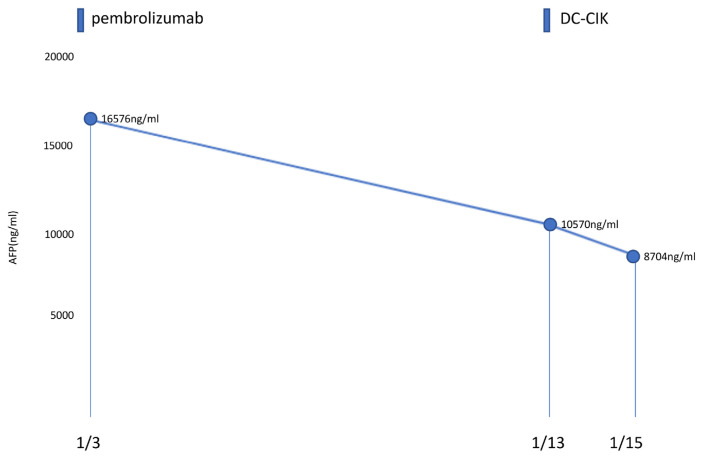
The serum AFP value during pembrolizumab and DC-CIK therapy. The AFP level decreased more rapidly after DC-CIK administration on 13 January 2022.

In this case, the decline of AFP level was 1.5 times more rapidly after DC-CIK administration during 13–15 January 2022 (933 ng/ml per day) compared to pembrolizumab administration during 01–13 January 2022 (600.6 ng/ml per day). Zibing Wang’s hypothesis might explain this phenomenon [15]. The initial infusion of pembrolizumab (half-life:23 days) [16] blocked PD-1 on new infused DC-CIK cell, leading to synergistic activity of therapy. Besides, increasing infiltration around the lung nodules after using cell therapy may be another evidence of the synergistic effect of immunotherapy [Figs. 2 and 3]. It is essential to mention that the AFP level dropped again after we discontinued methylprednisolone. The average AFP value decline from 24 January to 3 February 2022 was 727.2 ng/ml per day. The slope of this decline was similar to the one from 3 to 13 January 2022. The life span of dendritic cells and killer cells is about 14 days [17,18]. Therefore this effect might be attributed to pembrolizumab [Fig. 2].

The limitation of our case is the lack of a chest CT image and surgical intervention to know the leak site of his left lung. Besides, we did not use flow cytometry to analyze the patient peripheral blood mononuclear cell (PBMC) during the administration of pembrolizumab and DC-CIK vaccine.
